# Visual and Ocular Manifestations of Alzheimer’s Disease and Their Use as Biomarkers for Diagnosis and Progression

**DOI:** 10.3389/fneur.2016.00055

**Published:** 2016-04-19

**Authors:** Fatimah Zara Javaid, Jonathan Brenton, Li Guo, Maria F. Cordeiro

**Affiliations:** ^1^Glaucoma and Retinal Degeneration Research Group, Visual Neurosciences, UCL Institute of Ophthalmology, London, UK; ^2^Western Eye Hospital, Imperial College Healthcare NHS Trust, London, UK

**Keywords:** Alzheimer’s disease, neurodegereration, biomarkers, animal models of neurodegenerative disease, visual changes

## Abstract

Alzheimer’s disease (AD) is the most common form of dementia affecting the growing aging population today, with prevalence expected to rise over the next 35 years. Clinically, patients exhibit a progressive decline in cognition, memory, and social functioning due to deposition of amyloid β (Aβ) protein and intracellular hyperphosphorylated tau protein. These pathological hallmarks of AD are measured either through neuroimaging, cerebrospinal fluid analysis, or diagnosed post-mortem. Importantly, neuropathological progression occurs in the eye as well as the brain, and multiple visual changes have been noted in both human and animal models of AD. The eye offers itself as a transparent medium to cerebral pathology and has thus potentiated the development of ocular biomarkers for AD. The use of non-invasive screening, such as retinal imaging and visual testing, may enable earlier diagnosis in the clinical setting, minimizing invasive and expensive investigations. It also potentially improves disease management and quality of life for AD patients, as an earlier diagnosis allows initiation of medication and treatment. In this review, we explore the evidence surrounding ocular changes in AD and consider the biomarkers currently in development for early diagnosis.

## Introduction

The growth in life expectancy and the developing aging population has led to the increased prevalence of chronic diseases, such as Alzheimer’s disease (AD). Globally, there are almost 46 million people in the world living with dementia, with the number expected to rise to 131.5 million by the year 2050 (1). According to the World Health Organization (WHO) Global Burden of Disease (2004), dementia is the second largest contributor leading to total number of years living with disability (YLD) in people aged 60 years or older at 13.5%, compared to heart disease (4.0%), stroke (4.4%), and cancer (2.2%) (1).

Pathologically, AD is characterized by deposition of extracellular senile plaques, which is composed of amyloid β (Aβ) and intraneuronal neurofibrillary tangles (NFTs), resulting from intracellular aggregates of hyperphosphorylated tau protein detected in the brain (2). Aβ plaque deposition is associated with cross-sectional synaptic network dysfunction, progressive brain atrophy, and longitudinal cognitive decline (3). Studies have shown that Aβ and tau pathology correlates with neurocognition in mild cognitive impairment (MCI) (4). Less specific neuropathological lesions include granulovacuolar degeneration and eosinophilic rod-like bodies known as Hirano bodies (5).

Presently, AD can only be diagnosed post-mortem on histopathological examination. Diagnostic investigations are limited, and physicians rely on clinical examination and exclusion of differential diagnoses that may cause cognitive impairment, such as depression, Parkinson’s disease (PD), hypothyroidism, drug interactions, and vitamin deficiencies (6). Clinically, a diagnosis is made based on history, examination, and where available, accounts from relatives or carers. Nevertheless, premortem diagnoses have been considered inaccurate in 10–15% of cases even when assessed by experienced clinicians (7). Given the difficulties and delay in clinical diagnosis, patients often develop pathological damage prior to starting treatment. The advance of biomarkers using magnetic resonance imaging (MRI), positron emission tomography (PET), and cerebrospinal fluid (CSF) have led to the development of guidelines and diagnostic criteria (8–10). SPECT scanning can also be used to detect regional reduction in cerebral blood flow thought to be present in patients with AD (11).

Alzheimer’s disease is a heterogeneous disease and has multiple cognitive subtypes. These are usually broken down into memory, language, executive, attention, and visuospatial functioning (12). The variant of AD in which visual symptoms are prominent due to the localized pathology in the parieto-occipital region is often referred to as visual variant Alzheimer’s disease (VVAD) (13).

The interconnection between eye and brain suggests that it is reasonable to look for ocular manifestations of neurodegenerative disease and regard the eye as an extension of the CNS.

In embryological development, the eyes and brain have a similar origin. The eyes are formed from the anterior neural tube, an area that later gives rise to the forebrain. Ocular development occurs through specification of the eye field post-neural induction (14). This process involves specific transcription factors that are also conserved in brain development. One such factor, a “master regulator” gene of the development of the eye field, Pax6, plays an essential role in neural development. When expressed ectopically, Pax6 can induce ocular formation in other parts of the body (15), whereas its impairment or knockout disrupts neurogenesis in the cortex (16, 17).

In the eye, retinal neurons are comparable in many ways to their counterparts in the brain. Retinal neurons have dendrites, a soma and an axon, which are the essential neuronal features (18). They are stained by many typical neuronal markers (19–21). They form complex information processing networks similar to those in the cerebrum (22–24). The retina contains over 60 types of neuron (25) that play distinct roles in information processing. Photoreceptors represent one of the five main types of cells, the others being horizontal, bipolar, amacrine, and ganglion cell types. Photoreceptors transmit signals in a neuronal fashion when excited by light and are separated into two main subtypes, rods and cones. Bipolar cells transmit this information to retinal ganglion cells (RGCs). Horizontal cells are also connected to photoreceptors as well as bipolar cells and provide inhibitory feedback, to both adjust and refine the light signal. Amacrine cells can modify direct signals between bipolar and ganglion cells or can act as intermediaries between the two (26). In retinal networks, amacrine, bipolar, and horizontal cells act similar to interneurons, and the RGCs behave as projection neurons. Similar classes of neurotransmitters have been found in the retina, such as GABA and glutamate, which are essential to retinal information processing (27–29). Recently discovered group of retinal cell subtype are the intrinsically photosensitive retinal ganglion cells (ipRGCs). These cells respond to light through expression of the photopigment melanopsin in the absence of rod and cone photoreceptor input (30).

Therefore, due to its close association with the brain, it is not surprising that neurodegeneration caused by disorders such as Alzheimer’s extends into the eye. Visual symptoms have been well documented in AD, and there is significant evidence to illustrate that ocular pathology occurs as part of the disorder (31, 32). Consequently, this provides an opportunity to use a minimally invasive approach to examine the pathological features in the brain – through the transparent medium of the eye.

A critical component of research into AD is the use of animal models to standardize and replicate features of the disease in order to develop and assess response to treatments that would otherwise be unethical in human subjects. Approximately 98% of potential drug therapies fail in Phase 3 clinical trials, thus, pushing the focus of research toward targeting molecular pathways rather than symptom control (33). Animal models need to fit three validation criteria when used for human research (34). These are face validity, predictive validity, and constructive validity, which aim to ensure that animal model is based on relevant interpretation of the human condition.

With this in mind, many animal models have been proposed, including dog, rhesus monkey, *Drosophila melanogaster*, rat, and mouse (35). In particular, the mouse model is the most popular, as it has been noted to be most similar to human CNS structure in addition to its relatively inexpensive production of multiple transgenic strains (36). Transgenic mouse models of AD express single, double, or triple mutations found in familial AD. Genes encoding these mutations are presenelin 1 (PS1), presenelin 2 (PS2), tau, and amyloid precursor protein (APP). It is not the scope of this review article to detail all transgenic models available, but for the reader to have a brief background into the mutations involved.

There is a growing interest to identify a biomarker for AD to enable early diagnosis and prevent cognitive deterioration in patients. Genetic testing in AD has identified three genetic loci, *APP*, presenilin-1 (*PSEN1*), and presenilin-2 (*PSEN2*) as susceptibility genes for early-onset AD (EOAD) and SORL1 and APOE for late-onset AD (LOAD). Although these genes are useful in predicting the risk of developing AD, their lack of diagnostic specificity and sensitivity and the influence of external environmental factors make them unsuitable as biomarkers for AD (37–39). The majority of research has focused on the retina and associated changes in thickness, inflammation, and cell death. Nevertheless, manifestations of AD have additionally been identified in the pupil, lens, choroid, and optic nerve, though their use in the clinical setting has not been established (Table 1). Furthermore, changes in visual function have been identified in multiple studies (Table 2) and are also being investigated as potential indicators of AD pathology.

**Table 1:** **Manifestations of Alzheimer’s disease (AD) in the eye**.

Orbital structure	Pathological changes in AD
Pupil	Atypical pupil response to cholinergic antagonists (53, 54)
	Lower amplitude and latency of maximum reaction of pupillary light reflex (59)
	Increased pupillary size (62)
Lens	Aβ in lens and aqueous humor (66)
	Predisposition to supranuclear cataract (66)
Retina	Decreased retinal blood flow and RNFL thinning (75, 82, 83)
	RGC degeneration particularly in superior and inferior peripheral retina (100, 106)
	Overall reduction in RGC axon numbers (26)
	Aβ deposition in retina (94)
Choroid	Reduced choroidal thickness (101)
Optic nerve	Increased cup: disk ratio and pallor (148–150)

**Table 2:** **Visual manifestations of AD**.

Indicator of vision	Manifestation of AD	Recommended clinical test^a^
Visual acuity	Decreased visual acuity in low luminance (32, 33)	HOTV chart
Contrast sensitivity	Reduced visual contrast sensitivity particularly in low frequencies (37, 38)	Pelli–Robson chart (35)
	Reduced reading speed at lower contrast sensitivities (37)	Michelson contrast test (37)
Color vision	Poor color discrimination (43)	City University test
	Deficiencies most significant in tritan axis (31, 42)	Ishihara test (154)
Visual field loss	Inferior hemifield loss (45)	Humphrey automated perimetry (45)
		FDT (46)
Motion perception	Higher thresholds for motion detection across all spatial frequencies (57)	Computer animation sequences using random dot cinematogram (57)
Depth perception and stereopsis	Reduced stereopsis, mean threshold >150 s of arc	Randot stereotest (155)
Ocular motor function	Abnormal hypometric saccades	Eye movement examination (62)
	Increased latency as compared to controls (50, 51)	

*^a^All clinical tests require patient cooperation, which can be difficult in AD patients*.

*AD, Alzheimer’s disease; FDT, frequency doubling technique*.

## Visual Manifestations of AD

### Visual Acuity

Many studies have found no significant difference in visual acuity between AD patients and control subjects (40). Levels of luminance may affect visual acuity, as demonstrated in AD patients who had decreased visual acuity under conditions of low luminance (LL) (41, 42). A recent study has shown that AD patients have a significantly poorer accuracy and ability in recognition of pictures in LL and low spatial frequency (LSF), compared to age-matched controls (43). Another important factor to note in AD patients is the increased prevalence of cataract affecting visual acuity, which is addressed later in this review (44).

### Contrast Sensitivity

Contrast sensitivity allows for the ability to recognize objects over a range of spatial frequencies and is usually tested using charts (45) or electronic equipment. A deficit in this visual function greatly affects the daily functioning and quality of life and may explain the increased risk of falls and fractures in AD patients (46). AD patients have markedly reduced visual contrast sensitivity as compared to age-matched controls (47, 48) and are sensitive to testing even in early stages of AD. Furthermore, donepezil, an anticholinesterase inhibitor, has been shown to improve contrast sensitivity in AD patients (49). A reduction in reading speed has also been noted, particularly at lower contrast sensitivities (47). Studies have also shown that reading latency is increased in AD patients and is more evident with irregular words in text (50).

### Color Vision

Köllner first described changes in color vision, according to area of the eye affected, correlating retinal disease with blue–yellow visual changes and optic nerve disease with red–green visual loss (51). Earlier studies in AD patients have indicated that there are deficiencies in the tritan (blue) axis (40, 52), but more recent studies show no significant interaction in color axes (53). However, they do report an inversely proportional correlation with mini-mental state examination (MMSE) score and color discrimination error.

### Visual Field Loss

Visual field (VF) loss in AD is likely attributed to the neurodegenerative changes and synaptic dysfunction, particularly due to Aβ accumulation (54). Humphrey automated perimetry has been used in VF testing between AD patients and age-matched controls and has shown that the sensitivity losses occur particularly in the inferior hemifield of AD patients, and furthermore that degree of loss correlated with degree of dementia (55). VF testing with frequency doubling technology (FDT) also found that AD patients had VF deficits as compared to age-matched controls (56).

### Motion Perception

Motion perception is the process of deducing speed and direction of elements and is vital to everyday functioning and navigation. Studies have detected higher thresholds for motion detection in AD patients as compared to age-matched controls, and further correlation with dementia severity (57). Another recent study looked at visual motion processing and again found that patients with AD had higher thresholds across all spatial and temporal frequencies (58).

### Depth Perception and Stereopsis

Stereopsis is the perception of depth and 3D structure obtained from binocular vision. Studies have shown that patients with AD have reduced stereopsis as compared to control groups, and some also note a correlation between cognitive assessment scores and performance on stereopsis testing (59, 60).

### Ocular Motor Function

Alzheimer’s disease patients are known to have ocular motility dysfunctions and poor visual attention. They are often unable to focus on a fixed object, due to their difficulty in suppressing reflexive saccades. Crawford et al. used “eye tracking” software to assess saccadic eye movements in AD patients and found that they had slower reaction times as compared to the control group (61). Other studies have also shown patients with AD presented with abnormal hypometric saccades when tested, in addition to increased latency as compared to controls (62, 63). In particular, anti-saccades require movement of the eye in the opposite direction of a visual stimulus and suppression of the reflexive response. Functional MRI (fMRI) studies have shown patients with AD exhibited significantly more anti-saccade errors and, overall, demonstrated reduced activity in all oculomotor regions of the brain as compared to controls (64). The convergence angle has also been noted to be smaller and more irregular in AD patients (65).

## Pupil

It is now well established that patients with AD have an acetylcholine deficit and an altered ACh pathway (66). This became apparent in 1994 as a hypersensitive pupil response to cholinergic antagonist, tropicamide (0.01%), was discovered (67). AD patients’ pupils dilated to 13% more than controls. Subsequent experiments by Iijima et al. (68) found that significant differences in pupil sizes were found at a lower dose of tropicamide (0.005%), potentiating the possibility for a diagnostic screening test. Scinto (69) further investigated pupil involvement in AD and located a clear biological link between ApoE allele status and pupillary response to dilute tropicamide. Nevertheless, the use of the tropicamide test as a diagnostic tool for AD is controversial, as other studies have failed to find significant results (70–72).

Another indicator of AD pathology is the pupillary light reflex (PLR). Changes in the oculomotor system of the Edinger–Westphal nucleus and degeneration in the nucleus basalis of Meynert leading to cholinergic deficit are likely to lead to changes in the pupillary system. Pupillometry using image analysis technology can provide multiple parameters from a single PLR test, such as latency to pupil reaction, constriction acceleration and velocity, and amplitude of response. Prettyman et al. first described 75% latency of normal pupil size and the differences in constriction amplitude between AD patients and controls (73). Repetitive stimulation of the pupil light reflex over time is less pronounced in AD patients as compared to controls, with lower amplitude and latency of maximum reaction (74). Certain PLR measures have been found to correlate with cerebral plaque burden (75). Moreover, Bittner et al. found a significant correlation between increased pupillary size and CSF measures of both Aβ and tau (76). Color pupillometry has been used to measure the response of ipRGCs in various eye disorders (77, 78), but not yet tested in AD patients. This may be a potential biomarker requiring further investigation in future studies.

## Lens

Interest in the lens as an indicator of AD began after APP and Aβ expression were discovered in cultured mammalian lenses (79). In animal models, Aβ transgenic mice have developed lens opacification and shown to improve with EUK-189, a synthetic SOD/catalase, demonstrating a link between AD-associated cataracts and oxidative stress (80). Dutescu et al. also noted significant Aβ deposition in the lens of transgenic mice when labeling with WO2 and 1E8 antibodies, consistent with results from human studies (81).

In humans, Goldstein et al. detected the presence of Aβ in the lens and aqueous humor of AD patients (82). They also found that AD patients had a specific cataract in the supranuclear region not present in controls, which had high reactivity and staining against Aβ markers and antibodies. Similar cataracts have been found in Down syndrome patients, who have an increased level of Aβ due to a triplication of the APP gene (83). However, Michael et al. (84, 85) and Ho et al. (86) found no staining of Aβ in the lens of AD patients, although using different staining and characterization methodologies (84–86). Moreover, in a recent study, Bei et al. have concluded that opacities of the lens cannot be used as a non-invasive risk marker, as it does not vary significantly when controlling for age (87). Nonetheless, a clinical study by Kerbage et al. found that a fluorescent ligand for Aβ could be used *in vivo* to differentiate between AD patients and controls (88). There was a twofold greater signal in the AD patients compared to controls.

A subsequent clinical trial found the fluorescent signal of the same ligand to be able to distinguish between clinically diagnosed AD patients and controls with better results than a PET marker (89). This is a promising new development for diagnosing Alzheimer’s, although few studies have investigated changes during early stages of the disease. The incidence of cataracts and AD increases with age. This is important to note when using the lens as a biomarker for AD, as many patients will develop cataract irrespective of AD and, furthermore, may have undergone cataract surgery, thus limiting the use of the lens as a biomarker.

## Retina

### Retinal Vasculature

Similar to the brain, the retina has a highly isolated and thoroughly protected vasculature (90). Since there are vascular changes in the AD brain (91), it is likely that the analogous changes may be found in the AD retina. In a preliminary study, Berisha et al. found decreased vein diameter and decreased blood flow (92). In two recent large-scale studies (*n* = 456), AD patients had a less complex venular structure, smaller, more sparse, and tortuous retinal vessels (93, 94). Furthermore, recent studies have found significantly decreased retinal venous blood flow in AD patients (95). Importantly, MCI patients’ blood flow was found to be intermediate between AD patients and controls. This suggests that retinal blood flow (microliter per minute) might be used to monitor disease progression. They also observed that retinal nerve fiber layer (RNFL) thickness was decreased, but not significantly between AD patients and both MCI and control groups. This suggests that blood flow changes may precede cell death in the retina. Retinal oximetry is used to detect changes in eye metabolism and has been noted in a recent study to show abnormalities in AD. Einarsdottir et al. found that retinal oxygen saturation in arterioles and venules was elevated in AD patients as compared to controls (96).

### Retinal Thickness

Early histopathological studies implicated RGCs as the primary targets of cell loss in AD (97). As they demonstrated that the outer layers were relatively preserved in the postmortem retinas of AD patients, measurements have predominantly focused on the RNFL. Imaging technology, such as optical coherence tomography (OCT), has recently illustrated further evidence of RGC degeneration *in vivo* through measurement of the RNFL, indirectly evidencing ganglion cell loss (92, 98–100). An initial study by Parisi et al. found decreased RNFL thickness in all four retinal quadrants (101). RNFL thinning correlated with patients’ pattern electroretinogram performance, suggesting that RNFL thinning is related to visual dysfunction in AD. Current evidence also suggests that RNFL thickness decreases as the disease progresses and that there is a significant correlation between overall macula volume and level of cognitive impairment measured by the MMSE (102). Studies have shown significant differences not only between MCI patients and controls but also between MCI patient’s and two other AD groups (moderate and severe AD) in superior and total RNFL thickness (103). A recent meta-analysis (104) of 11 AD OCT studies found significant reduction in mean RNFL and in all 4 individual retinal quadrants around the AD macula. In three of these studies containing MCI patients, decreased RNFL thickness was found compared to controls. Bambo et al. also noted significant RNFL thinning in the superior and inferior quadrants of AD patients (105).

A few animal studies have published similar findings with regards to neuronal cell loss in the RGCL. One study using a single transgenic mouse model reported a statistically significant decrease in the retinal thickness of the Tg2576 mice from the GCL to the ONL as compared to wild-type control animals (106). This suggests that retinal degeneration may affect all retinal cell types and layers.

Recently, Ong et al. (107) combined the use of OCT and MRI scanning in an elderly population (>60 years old). They discovered that decreases in GC-IPL thickness correlated with decreased size of the occipital and temporal regions of the brain. These correlations suggest that degeneration in the retina is paralleled in specific regions of the brain, which are implicated in AD.

### Aβ in the Retina

Synaptic dysfunction and neuronal cell death because of Aβ toxic deposits are pathological hallmarks of AD (54). In animal models, overexpression of the human APP Swedish gene in Tg2576 mouse results in deposition of Aβ plaques in the brain (81). This strain of mice developed memory deficits at 10 months of age as compared to the APPswe/PS1-del9 transgenic strain that developed deficits earlier at 6 months of age (108). Aβ plaques have since been reported in the same strain, from the ganglion cell layer to the inner plexiform layer, in addition to plaques found in the outer segment and optic nerve (106, 109) (Table 3). Thus, this model suggests that ganglion cells and potentially retinal interneurons (horizontal, bipolar, and amacrine cells) are affected by Aβ plaques; yet, all retinal cells may be compromised in later stages of the disorder.

**Table 3:** **Location of APP and Aβ found in the animal retina**.

Retinal layer	APP	Aβ	Tau
Retinal pigment epithelium	−	−	−
Outer nuclear layer	−	+	−
Inner nuclear layer	+	+	−
Inner plexiform layer	+	+	−
Ganglion cell layer	+	+	+

*+Present*.

*−Absent*.

*APP has been found in the ganglion cell layer through the inner nuclear layer in AD animal models (65, 81, 86, 106). Aβ has been found in the retina in all layers apart from the retinal pigment epithelium (81, 88, 93, 109, 114). Tau has been found in ganglion cell layer (99, 106, 133)*.

Amyloid β loads are further associated with immunoreactivity for MCP-1, F4/80, and TUNEL-positive profiles in the RGC layer in mutant presenilin (PS1) and APP transgenic mice. This further indicates that Aβ deposition causes retinal neurodegeneration in mouse models of AD (110). Transgenic mice overexpressing the Swedish mutation (APP23) and mutant human APP and mutant human presenilin-1 (APPPS1) exhibit a threefold increase in total endogenous murine Tau in CSF and an age-related increase in Aβ deposition (111).

*In vivo* imaging in APP/PS1 transgenic mice following administration of systemic curcumin has provided a potential tool for monitoring Aβ plaque formation in AD (112). Optical imaging showed increased plaque formation with age in the mouse model, and remarkably, a decrease in response to glatiramer acetate immunotherapy. APP and Aβ have both been located in the human retina (113); however, these were not consistently found (32). A major advance was made in 2011 in human AD, where Aβ was visualized in the postmortem retina and also live animal retina (114). Very recent work has revealed substantial Aβ deposition in postmortem AD retinas and that these deposits may preferentially target the melanopsin-staining subtype of RGC (115).

Amyloid β has been identified in retinal drusen, a hallmark of age-related macular degeneration (AMD), a major cause of worldwide blindness. Drusen are abnormal extracellular deposits along the basal surface of the retinal pigmented epithelium (RPE). Aβ-containing drusen are associated with RPE atrophy and photoreceptor death (116, 117). In AMD, they are predominantly located in the macula at the center of the retina, but they can still occur peripherally. In fact, peripheral drusen have been found to be significantly associated with AD (118, 119).

It is believed that Aβ can enter the RPE through advanced glycation end products (RAGE)/p38 MAPK-mediated endocytosis. Intracellular Aβ triggers breakdown of RPE tight junctions (120), as described in Tg AD mice (121). The toxic effects of Aβ on RPE include reduced mitochondrial redox potential and increased reactive oxygen species (ROS) production, RPE pigmentation and hypertrophy, followed by photoreceptor death (122). In addition, Aβ-mediated gliosis of Müller cells (MCs) has been implicated in retinal degeneration. MCs are the principal retinal glial cells and metabolically coupled to photoreceptors. Glial cell activation in response to Aβ deposition has compromised the integrity of the blood–retina barrier, leading to photoreceptor apoptosis (123). Interestingly, Aβ did not induce cell death in purified photoreceptor cell cultures but in mixed retinal cell cultures, suggesting that the cellular environment plays a role in Aβ-mediated photoreceptor apoptosis (124). Furthermore, Aβ has been implicated in complement activation by upregulating factor B, the main activator of the complement alternative pathway, in RPE through cytokines, which are released from recruited macrophages/microglia (125). This may explain the colocalization of Aβ with activated complement components found in some drusen (126, 127). Similar observations have also been reported in senile Aβ plaques in the brain, where Aβ is thought to be a primary activator of complement in AD (128), suggesting common mechanisms may be applied to AD, drusen formation, and AMD.

### Tau in the Retina

Tau accumulation has been observed in brains of doubly transgenic mice from 4.5 months of age (129) and in the hippocampus and amygdala of triple-transgenic mice (130). In single Tg2576 transgenic mice (APP), hyperphosphorylated tau was observed in adjacent sections of Aβ deposition in the ganglion cell layer (106). Double-transgenic mice APP/PS1 also displayed hyperexpression of tau in the retina with consequent upregulation of p35, p25, and calpain, which has been widely hypothesized to cause synaptic dysfunction and calcium dysregulation in the context AD-related apoptosis (131, 132). In human P301S tau transgenic mice, tau aggregates formed in the RNFL resulting in axonopathy and at 2 months of age formed tau inclusions within RGC (133). Tau has been found in the human retina (113); yet, this finding has not been consistently replicated (86, 134).

### Retinal Fluorescence and Neurodegeneration

In animal models, retinal changes observed in double-transgenic mice included accumulation of Aβ peptides in addition to detection of apoptotic cells in the RGC layer (135). Another study using the double-transgenic model (Tg2576 × Tg1) observed that 27-month-old mice had a 200% increase in the number of apoptosing cells in the GCL, as compared to 7.8-month-old mice (110).

A more extensive triple-transgenic mouse model expressing human APP, PS1, and tau mutations was used to investigate retinal glial changes in AD. The study by Edwards et al. found that at 9 months, MCs were activated, and astrocytes increased in size and number indicating retinal glial activation (136). Further evidence for involvement of the outer retina in AD models of disease is depicted in transgenic AD rat models. Tsai et al. found on staining of retinal sections that RPE cells showed marked hypertrophy and frequently contained two nuclei rather than one (137).

*In vivo* imaging has recently developed to allow direct visualization of apoptosing cells in the retina. A novel technique has been established using radiolabeled annexin V and confocal laser scanning ophthalmoscopy to detect cell apoptosis in the retina (138). This *in vivo* imaging method is known as detection of apoptosing retinal cells (DARC). Using this imaging technique and applying it to animal models provides further evidence of RGC apoptosis in AD. Following PI and annexin-IR intravitreal injection in triple-transgenic AD mice, a significant number of RGCs were observed to undergo early-phase apoptosis as compared to age-matched controls (139).

A small pilot study using retinal fluorescence lifetime imaging ophthalmoscopy (FLIO) suggested that retinal changes detected by FLIO correlated with clinical characteristics of AD and could potentially be used as a biomarker for AD. However, the lack of control group and small study size warrants further research before attributing these general changes specifically to AD (140). Another study used systemic curcumin administration followed by optical imaging to successfully label retinal Aβ plaques in mice (114). This has also been trialed in patients using curcumin supplements and retinal fluorescence imaging (141). Furthermore, recent research has established methods of identifying drusen deposits in the peripheral retina using ultra-wide-field imaging (142, 143). This is particularly of note in patients with AD, as these small deposits were not found in age-matched controls, thus providing possibility for another potential biomarker for diagnosis of early AD.

## Choroid

Most research has been directed toward retinal changes in AD given the direct association with the brain. However, a few studies have observed possible choroidal manifestations of AD in animal models. Heterozygous transgenic rats (TgF344-AD) had significantly reduced choroidal thickness as compared to age-matched controls (137). Also, of note in this particular study was the upregulation of complement factor C3, which has previously been noted to play a role in RGC related apoptosis, studied particularly in glaucoma (144). Ning et al. also reported age-dependent Aβ deposits in the retinal and choroidal vasculature of two strains of transgenic mice (110).

In human models, recent imaging of the choroid in AD patients showed decreased choroidal thickness (145). However, given the limited research undertaken, more studies are needed to confirm if the choroid is indeed damaged in AD.

## Optic Nerve and Neurodegeneration

Cell death of RGC and changes in the optic nerve head have long been noted in postmortem AD retinas and first described by Hinton et al. who found a variety of degenerative profiles in RGCs, including cell shrinkage and cell swelling with vacuolization (97). Moreover, there was a two to threefold reduction in RGC axon numbers compared to controls. Further research (146) found the same features of degeneration in addition to nuclear fragmentation and heavily silver-stained cytoplasm. It was also noted that AD optic nerves had approximately half the RGC axon density of controls (32). This suggests that the larger “M” cell type RGCs are chiefly affected by AD. Blanks et al. found a 25% reduction of RGCs in the central 3 mm of the AD retina and severe cell loss over the entire retina (134, 147). This degeneration is more pronounced in the superior and inferior peripheral regions.

More recently, new imaging technologies have provided *in vivo* evidence of optic nerve head pathology. Multiple studies, using OCT and confocal laser scanning, have found greater cup-to-disk ratio and increased pallor of the AD optic nerve, representing a significant loss of RGC axons (148–151).

## Future Implications

As outlined earlier in this review article, specialist brain imaging modalities, such as fMRI and PET scanning, can support a diagnosis of AD. Unfortunately, these are expensive investigative techniques and are often limited to a hospital setting. Furthermore, changes in the brain are often a sign of later-stage AD and symptoms of cognitive decline will already have set in. Remarkably, studies now support the hypothesis that Aβ plaques appear earlier in the retina (114) and so propose the possibility for earlier diagnosis of AD and subsequent treatment.

Alzheimer’s disease-related retinal degeneration has provided a novel technique for investigating AD pathology and targeting treatment. Multiple human and animal studies have illustrated the correlation of AD neuropathology in the retina and the brain, including Aβ and tau accumulation, neuronal cell loss, and RGC apoptosis. Using the eye and its transparent medium as an “extension of the brain” allows for non-invasive visualization of AD pathology *in vivo*, and enables monitoring of biomarkers in order to diagnose and potentially monitor development and treatment of AD.

Technology, such as DARC, has provided a platform for this (139), and different approaches to *in vivo* imaging are constantly evolving (114, 143, 152). Unfortunately, these methods of *in vivo* imaging are not without limitations, particularly with regards to specificity for Aβ plaques. Currently, the use of OCT to assess the ONH and RNFL are useful in detecting neurodegeneration, in addition to a comprehensive eye examination in the clinical setting covering VF defects, pupillometry, and contrast sensitivity. *In vivo* imaging is emerging as a potential biomarker in many neurodegenerative diseases (138, 153), and the evolution and development of such imaging techniques today prove promising as diagnostic tools of the future.

## Author Contributions

FJ: preparation of manuscript, editing, proofing and submitting author; JB: preparation of manuscript, editing; LG: editing manuscript; MC: overall editing and corresponding author.

## Conflict of Interest Statement

The authors declare that the research was conducted in the absence of any commercial or financial relationships that could be construed as a potential conflict of interest.
